# Foul tip impact attenuation of baseball catcher masks using head impact metrics

**DOI:** 10.1371/journal.pone.0198316

**Published:** 2018-06-01

**Authors:** Christopher P. Eckersley, Terrance R. White, Hattie C. Cutcliffe, Jay K. Shridharani, Garrett W. Wood, Cameron R. Bass

**Affiliations:** Department of Biomedical Engineering, Duke University, Durham, North Carolina, United States of America; University of Innsbruck, AUSTRIA

## Abstract

Currently, no scientific consensus exists on the relative safety of catcher mask styles and materials. Due to differences in mass and material properties, the style and material of a catcher mask influences the impact metrics observed during simulated foul ball impacts. The catcher surrogate was a Hybrid III head and neck equipped with a six degree of freedom sensor package to obtain linear accelerations and angular rates. Four mask styles were impacted using an air cannon for six 30 m/s and six 35 m/s impacts to the nasion. To quantify impact severity, the metrics peak linear acceleration, peak angular acceleration, Head Injury Criterion, Head Impact Power, and Gadd Severity Index were used. An Analysis of Covariance and a Tukey’s HSD Test were conducted to compare the least squares mean between masks for each head injury metric. For each injury metric a P-Value less than 0.05 was found indicating a significant difference in mask performance. Tukey’s HSD test found for each metric, the traditional style titanium mask fell in the lowest performance category while the hockey style mask was in the highest performance category. Limitations of this study prevented a direct correlation from mask testing performance to mild traumatic brain injury.

## Introduction

In the last decade, concussions have surfaced as a common problem within sports and recreational activities. Within the United States, there is an estimate of 1.6 to 3.8 million cases of sports-related traumatic brain injuries annually[1]. Defined by the 1996 Committee of Head Injury Nomenclature, a concussion is known to be “a clinical syndrome characterized by immediate and transient post-traumatic impairment of neural functions, such as an alteration of consciousness, disturbance of vision or equilibrium due to brain stem involvement.” Concussions are accompanied by the difficulty of thinking clearly or concentrating, headaches, nausea, dizziness, blurry vision, fatigue, and balance problems[2–4].

**Fig 1:**
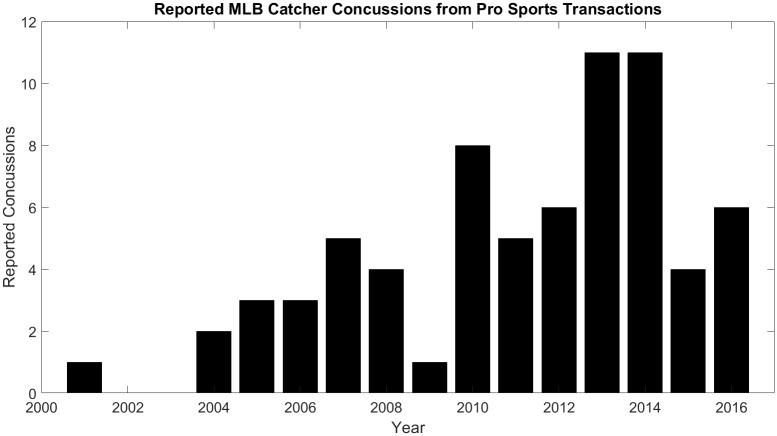
Bar plot of reported concussions in MLB catchers shows general increasing trend since 2000.

As seen in Fig 1, there has been an increasing trend in the number of concussions reported by Major League Baseball (MLB) catchers, and a majority are due to foul tip impacts[5]. This trend could be due to a number of factors including increased concussion awareness and increased pitching velocity. However, concurrent with this increasing trend has been the introduction of novel catcher mask technology and styles, which is believed to play an influential role in the reported concussion increase. In 2006 Nike introduced a mask made of titanium that was significantly lighter than the traditional steel masks. In 2013, ten of the reported concussions by MLB catchers were caused by foul tip impacts, and five of those catchers were wearing a titanium mask.

However, the controversy extends beyond titanium masks; several catchers have claimed in the media one mask is safer than another, however some of their reports contradict each other. These claims have been made although little is known about the relative ability for different catcher mask models to reduce concussion risk. To date, a study has not been conducted to compare the wide variety of catcher mask models on the market using various impact metrics. In 2010, Shain et al. analyzed the ability for catcher masks to attenuate impacts compared to a bare head condition, and while two masks were used, not enough data was obtained to make a comparison between masks[6]. Beyer et al. in 2012 looked at the effect of impact location on head accelerations for the hockey style and traditional steel catcher masks from both on field data and experimentation. They determined that there was little variation between the mask types and that impacting the eyebrow and chin resulted in the highest linear and angular accelerations[7]. In 2014, Laudner et al. compared head accelerations between a traditional steel mask and a Wilson Shock FX 2.0 hockey style mask for locations around the entire head. It was determined that for front impact scenarios impacting the mask, there was not a statistically significant difference in head accelerations between masks[8]. They also tested impacts to the plastic component of the helmet where they did find a statistically significant difference, although that scenario is not analyzed in this study. Finally, in 2016, Siu et al. compared the linear acceleration response of a hockey style mask, steel traditional style mask, and modified traditional style mask at four locations and three impact angles. It was determined for impacts closest to the nasion region, there was not a significant difference between the traditional and hockey style masks[9].

The goal of this study was to investigate the impact response of four styles of catcher masks (traditional steel, traditional titanium, hockey style, and hockey style with shock adsorption). Mask comparison and impact attenuation were assessed using five impact metrics: peak linear resultant acceleration, peak angular resultant acceleration, Head Injury Criterion (HIC15), Head Impact Power (HIP) and Gadd Severity Index (GSI). It was hypothesized, that due to decreased mass and increased yield strength, the titanium mask will prove the least effective at minimizing head impact metrics. Conversely, due to their increased mass the two hockey style masks will prove to be most effective at minimizing head impact metrics.

## Materials and methods

**Fig 2:**
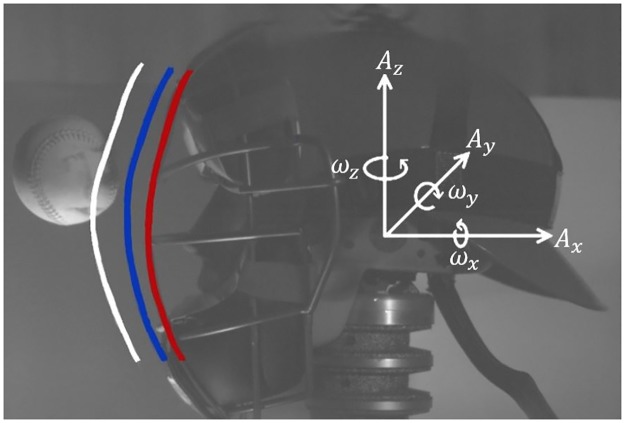
Illustration of mask deformation distance and coordinate axis used for linear accelerations and angular rates. White Line—Initial position of mask no deformation, Red Line—Final position of mask maximum deformation.

Data for this study was collected in October 2015. For a catcher surrogate, a Hybrid III head and neck were rigidly bolted to a raising platform. While this differs from several similar studies that position this setup on a slide table, it is believed to not influence the results because the neck decouples the head from the mount during the short duration impact event. The head was equipped with a six degree of freedom sensor package mounted at the heads center of gravity. The sensor package consists of three piezoresistive linear accelerometers (Endevco, USA) oriented orthogonally, along with three angular rate sensors (DTS, USA) to produce the coordinate axis seen in Fig 2. The mask was positioned on the head after every trial to ensure it is located on the face according to manufacture specifications.

**Fig 3:**
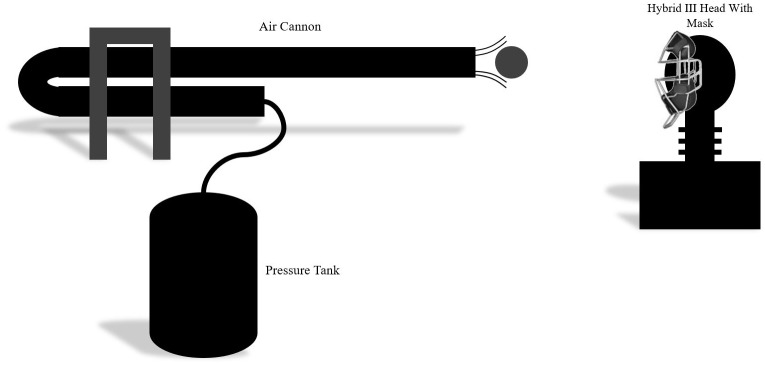
Illustration of the test setup. Air cannon made of 3 in, schedule 40 pipe with 1.52 m barrel placed 1.5 meters from the front of the mask.

A PVC air cannon made of 3 in, schedule 40 PVC pipe with a 1.52 m long barrel and 0.91 m long air chamber connected with a quick release valve was used to propel Rawlings Official Major League baseballs (Rawlings, USA) that have a mass of 142 g and diameter of 75 mm. The cannon was positioned approximately 1.5 m from the head setup and pressurized to 344 kPa and 413 kPa. These pressures were selected based on a generated calibration curve; pressures of 344 and 413 kPa correspond to 30 and 35 m/s pitch velocities at the moment of mask impact respectively. The velocities were desirable since they were low enough to prevent rapid catastrophic mask destruction, but were still in a range relevant to on field scenarios and studies previously conducted[6–9]. Fig 3 illustrates the cannon and Hybrid III setup.

**Fig 4:**
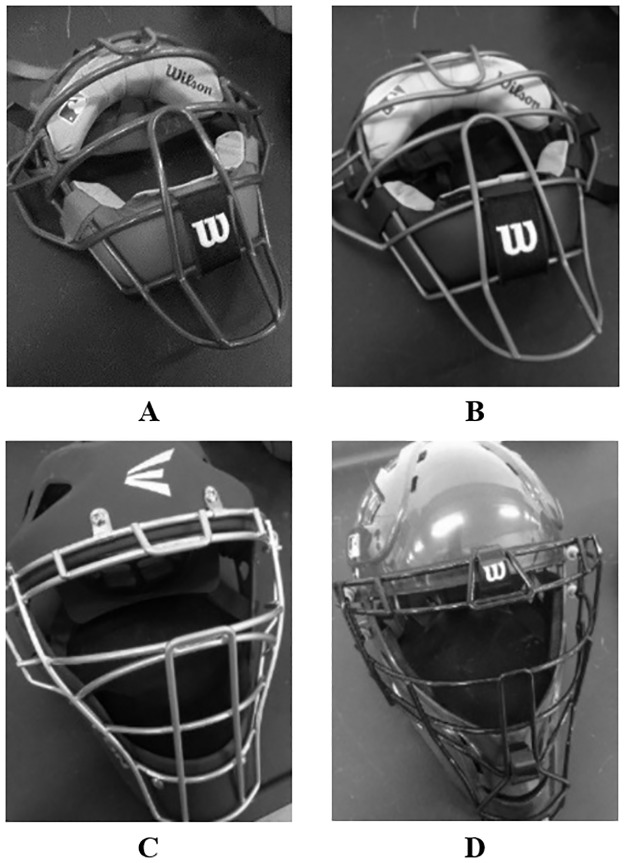
Images of catcher masks used. (A) Wilson Dynalite Traditional Steel, (B) Wilson Dynalite Traditional Titanium, (C) Easton Rival Hockey Style, (D) Wilson Shock FX 2.0 Hockey Style.

In this study, four catcher mask models were analyzed, the Wilson Dyna-Lite traditional steel mask with cap, the Wilson Dyna-Lite traditional titanium mask with cap, the Easton Rival hockey style mask with a steel cage, and the Wilson Shock FX 2.0 hockey style mask with a steel cage. Fig 4 shows images of each mask and Table 1 provides the mass of each mask.

**Table 1:** Mass of each catcher mask.

Mask	Mass (g)
Steel Traditional (w/ Cap)	726 (1063)
Titanium Traditional (w/ Cap)	567 (903)
Hockey	1113
Shock FX	1190

The Wilson traditional style masks were selected as they allow for a direct material comparison between titanium and steel since they have the same design and padding. The Easton Rival hockey mask was chosen for its common use as a hockey style mask, particularly in populations where hockey style masks are required. Finally, the Shock FX 2.0 was selected since it uses one of the newest technologies on the market, and claims to have impact shock adsorbing capabilities of up to 50%. However, it is not known how that translates to reducing head accelerations.

For each mask, twelve trials were conducted, first six at 344 kPa then six at 413 kPa, resulting in forty-eight total trials. This resulted in pitch velocities of 30.9±0.4 m/s and 35.7±0.5 m/s. A power analysis showed that given the estimated standard deviation of 3.5, twelve trials are able to detect an effect size of four with 0.8 power. Each trial impacted the head centered on the face just below the eyebrow in the nasion region. This position was selected based on work by Beyer et al. that found that the forehead and chin region resulted in the highest angular accelerations[7]. While the nasion is slightly below the forehead, contacting this location resulted in a more direct impact ensuring maximum energy transfer from the ball into the mask head system. For each trial, data was sampled at 200 kHz using a MeDAQ acquisition system (Hi-Techniques, USA) and linear accelerometer and angular rate data were filtered according to CFC 1000 and CFC 180 respectively[10]. The velocity of each pitch and qualitative analysis of impact was determined using high speed video footage captured by a Phantom High Speed Camera at 200 kHz (Vision Research, USA).

Peak resultant linear acceleration, peak resultant angular acceleration, HIC15, HIP, and GSI were the head impact metrics chosen to compare the masks. Peak resultant linear acceleration is a metric commonly used to easily describe the accelerations experienced by a given impact, and has been extensively study in relation to head injury[6–8]. Peak resultant angular acceleration was selected based on work published by Eucker et al. Rotational inertial forces are believed be the underlying mechanism in Traumatic Brain Injury (TBI) and likely also contribute to mild Traumatic Brain Injury (mTBI)[11]. HIC15 was first developed for use in the automotive safety industry as a metric to develop injury criterion for head impacts[12–14]. It is found using Eq 1 and utilizes the integral of linear acceleration. This is thought to be a useful metric since it takes into account both the acceleration magnitude and duration. HIP is an injury metric recently developed in an effort to determine a more effective injury criterion and is found using Eq 2 where all accelerations and velocities are functions of time[15, 16]. The strength of HIP is that it not only includes aspects of linear and angular velocity and acceleration, but also weights each of the components based on head geometry. Finally, GSI is currently used by the National Operation Committee of Standards for Athletic Equipment (NOCSAE) to certify catcher masks for use on the public market[17]. It is calculated using Eq 3, and values from this study will be compared to current certification criterion[18].

$$HIC\;=\;{\left\{{\left[\frac{1}{t_{2}-t_{1}}\int_{t_{1}}^{t_{2}}A_{res}(t)dt\;\right]}^{2.5}\right\}}_{MAX}$$ (1)

$$HIP\;=\;4.5(A_{x}V_{x}+A_{y}V_{y}+A_{z}V_{z})+0.016\omega_{x}\alpha_{x}+0.024\omega_{y}\alpha_{y}+0.022\omega_{z}\alpha_{z}$$ (2)

$$GSI\;=\;\int_{t_{1}}^{t_{2}}A_{res}{\left(t\right)}^{2.5}dt$$ (3)

Each head injury criterion was calculated from the linear acceleration and angular rate data using a custom MATLAB analysis suite (MathWorks, USA). For this study, the independent variable was mask type, the dependent variable was the injury metric, and the covariate was the velocity of the ball. Statistical analysis using JMP Pro (SAS, USA) consisted of initially conducting an Analysis of Covariance (ANCOVA) with an interaction between the independent variable covariate for each injury metric to test the homogeneity of regression. If the homogeneity of regression criterion was met, then the ANCOVA was conducted without the interaction. If the null hypothesis was rejected by the ANCOVA test (*α<* 0.05), Tukey’s HSD Test was conducted on the data to compare the least squares mean between each mask combination. Both the ANCOVA with and without an interaction accounted for unequal group sizes when comparing the difference between regression lines, and significance was determined using a Type III sum of squares. An ANCOVA was selected as the optimal statistical analysis because each impact is treated as an independent event and the velocity is treated as a continuous variable to account for its larger variation. The events can be assumed to be independent because in-between each test, the mask was repositioned to its original state, viscoelastic materials were given time to return to their original conditions, and the influence of any plastic deformation that may have occurred was recorded and not found to influence final results as long as the ball did not make contact with the head.

All raw data collected for this study has been published in the Duke Digital Repository with DOI 10.7924/G81N7Z29 and can be found at https://dx.doi.org/10.7924/G81N7Z29.

## Results

Twelve trials were attempted for each mask, however only eleven and seven of the trials for the steel and Shock FX 2.0 masks respectively were successful. On one of the 30 m/s trials for the steel mask, a cannon malfunction led to a low ball velocity and the outlier was removed from the data set. The Shock FX 2.0 mask experienced significant damage during trials. High speed video analysis showed that for one of the 30 m/s and four of the 35 m/s tests, the mask did not prevent the ball from impacting the head. Those trials resulted in higher head accelerations and were also omitted from the data set. These trials could not be repeated due to the plastic deformation of the masks.

**Fig 5:**
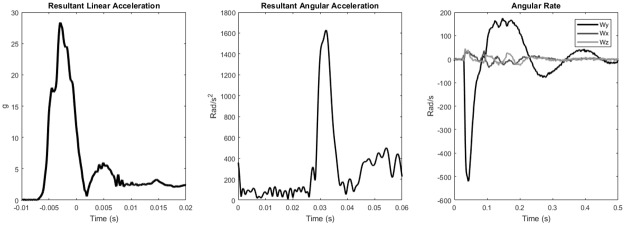
Representative traces of resultant linear acceleration, resultant angular acceleration, and angular rate to show relative impact duration and magnitude.

Acceleration data shows the main linear acceleration event occurs over the span of 10 milliseconds with the peak occurring approximately 5 milliseconds after impact. Similar to the linear acceleration, the resultant angular acceleration occurs over approximately a 10 millisecond interval. Fig 5 depicts representative linear acceleration, angular acceleration, and angular rate traces.

**Fig 6:**
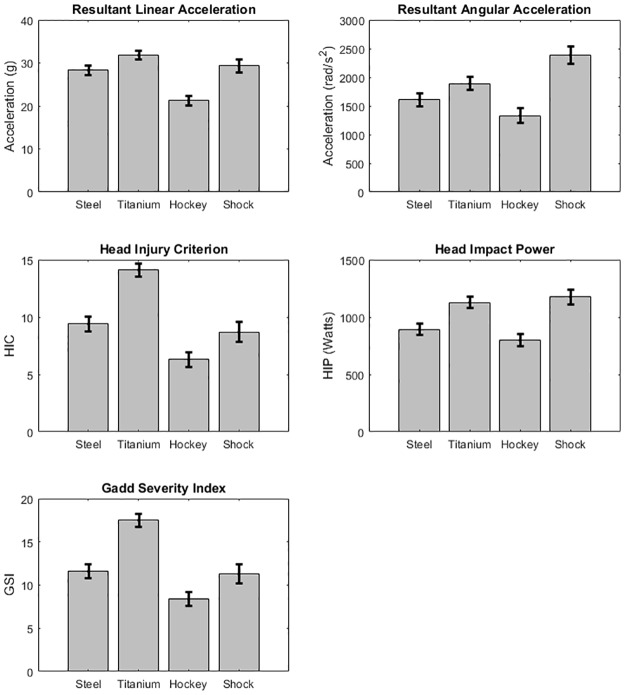
Bar plots of the least squares mean for each impact metric and each mask type. Error bars are one standard error from the mean.

For resultant linear acceleration, HIC15, and GSI, the interaction between velocity and mask type was statistically significant, meaning the masks responded differently to changes in velocity. Specifically, the shock mask had a smaller injury metric increase between 30 m/s and 35 m/s than the other three masks. The ANCOVA tests found statistical significance to reject the null hypothesis for every head injury metric. Fig 6 shows the least squares mean plots for each of the impact metrics with error bars representing one standard error. The results of Tukey’s HSD tests for each head injury metric are summarized in Table 2, with lower numbers indicating lower head injury metric values. Statistical calculations show that the traditional style titanium masks fell in the lowest performance category while the hockey style mask was in the highest performance category for each injury metric.

**Table 2:** Summary of results from Tukey’s HSD test.

		Head Impact Metrics				
		Res. Lin.	Res. Ang.	HIC15	HIP	GSI
**Mask Type**	**Hockey**	1	1	1	1	1
	**Shock**	2	2	3	2	1,2
	**Steel**	2	1,2	1,2	1	2
	**Titanium**	2	3	2,3	2	3

Lower numbers indicate lower head injury metric values

## Discussion

The primary motivation behind this study was to examine how the foul tip impact attenuation of catcher masks was affected by style and material. Statistical analysis shows that wearing the titanium mask resulted in the highest values for each head injury metric. This supports the hypothesis and is most likely due to two factors, mass and yield strength. Since the titanium mask is lighter, the overall head mass will be less and will result in higher accelerations for a given input force. The yield strength of titanium is significantly higher than that of steel (1.4 GPa vs. 0.4 GPa) meaning that for a given force, the mask will experience less plastic deformation upon impact and absorb less energy. Statistical analysis also found that the hockey style mask resulted in the lowest values for each head injury metric. This makes sense due to the mask’s increased mass which will help lower head accelerations, but since it has a comparable mass to the Shock FX mask this cannot be the only factor. The interior of the Easton Rival hockey style mask is lined with an energy absorbing viscoelastic foam, while all three Wilson models are lined with a more elastic foam. It is not known quantitatively how much of an effect the viscoelastic nature of this particular foam has on head accelerations, but it has been shown that foams of different types and stiffness can influence the impact kinematic response[19].

The significant interaction between velocity and mask type proposed the shock mask increases its performance compared to the other masks as velocity increases. This could be due to a number of reasons, first is the viscoelastic properties of the shock absorbers may be optimized for high velocity impact and may be less at lower 30 m/s velocities. Second due to damage only 2 trials were conducted at higher velocities, which may not be enough to capture the true impact response of the mask.

Shain et al. were the first group to analyze the effectiveness of catcher masks to attenuate foul ball impacts. The masks analyzed by Shain et al. were a hockey style and traditional steel mask. For each of the metrics derived from linear acceleration (Peak Resultant Linear, HIC15, and GSI) the data from this study and Shain et al. are consistent and, as discussed in Shain et al., are below common injury thresholds currently used. However, the angular acceleration data by Shain et al. are higher than those in this study. This is most likely due to two factors. The first is how angular accelerations were calculated; Shain et al. derived the angular acceleration values from a 3-2-2-2 linear accelerometer setup filtered according to CFC 1000 while this study differentiated angular rate data filtered at CFC 180 to obtain angular acceleration[10]. Second, Shain et al. impacted the mask slighter higher in the Z direction than in this study, and as seen in Beyer et al. the vertical location of impact can have an effect on the angular accelerations measured.

Beyer et al. conducted an analysis of several impact locations utilizing traditional steel and hockey style masks in effort to determine if impact location has an effect on head accelerations. This allows for data comparison of similar impact locations between Beyer et al. and this study. The linear accelerations have comparable values while the angular accelerations determined by Beyer et al. are higher than those in this study. There are two key differences between the datasets; Beyer et al. used the same angular acceleration derivation method as Shain et al. and tested higher average velocity than the data in this study. However, since Shainet al. tested average velocities lower than this study, and Beyer et al. tested a similar location, but both had higher angular accelerations, the most significant factor in the difference between angular acceleration values is believed to be the method of derivation and filtering.

Laudner et al. also analyzed location of impact, but the important aspect in relation to this study is that for front impacts, the difference in accelerations between the traditional steel mask and the Shock FX 2.0 is not statistically significant. This is also reflected in the results of this study and is important since the manufacture claims that the Shock FX 2.0 absorbs up to 50% of the energy from an impact compared to traditional masks. While these are not definitive measurements of energy, it has been shown in two studies that the presence of the shock absorbing mechanism has little to no effect on minimizing head accelerations.

**Fig 7:**
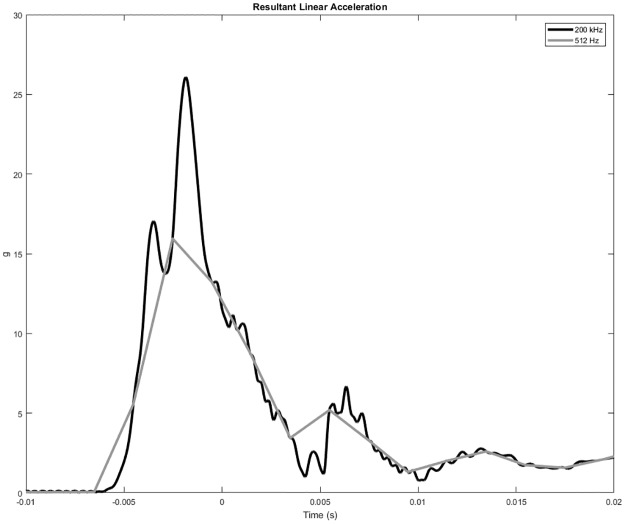
Comparison of linear acceleration trace sampled at 200 kHz down sampled to 512 Hz.

Much like Beyer et al. and Laudner et al., Siu et al. analyzed a variety of impact locations and directions. For head on impacts to the nasion region, they did not find a statistically significant difference between the transverse peak linear acceleration of the hockey style and traditional style masks. This contradicts the results found in this study which can be attributed to several differences. The first is that Siu et al. sampled at a frequency of only 512 Hz, which is too low a resolution to accurately capture the linear acceleration of a catcher mask impact. Fig 7 compares data collected in this study, sampled at 200 kHz, to a down sampled version at 500 Hz. Second, the model used requires validation of head and neck kinematics in the transverse plane. The model used by Siu et al. most likely has different viscoelastic properties compared to the Hybrid III head and neck, and variations in global viscoelastic properties may have a large influence on the system’skinematic response.

According to NOCSAE DOC .024, which was last updated in 2015, in order for a catcher mask to be certified for public use, it cannot result in a GSI value greater than 1200 during the testing battery[17]. This is a large value when compared to the maximum GSI value of 30.9 experienced during this study. This is an issue since masks that result in injury on the field can still easily earn safety certification. There are several explanations for this discrepancy. First, the standard was develop to prevent catastrophic head injury; the standard has proven effectivefor this injury but may need to be adapted as the prevalent injury changes. Second, the value of 1200 was chosen based on the current standard for football impacts, which only includes a drop test. The catcher mask standard includes both a drop test and a projectile impact. A GSI of 1200 is not suitable for a projectile impact, because the energy inputs are different. Drop tests impact the near infinite mass of the earth, while projectile impacts incorporate the finite mass of a projectile. Furthermore, the NOCSAE standard uses a rigid neck attachment while this study utilizes a Hybrid III neck. The Hybrid III neck increases the compliance of the system and lowers GSI values. Finally, GSI is believed to be a poor predictor of head injury as it does not account for rotational acceleration, and like HIC15 was developed for severe injuries (e.g. skull fracture) in the automobile industry, which do not directly correlate to mTBI[20].

This study illustrates the dichotomy between athlete performance and athlete safety that often plagues sports safety equipment manufacturers. Based on the results of this study, it would be optimal from a safety perspective to make masks as large and heavy as possible. However, if this were the case, no athlete would wear the mask because it would hinder their performance. There is a balance between safety and performance that needs to be optimized.

There were a number of limitations that arose when conducting this study. Only one brand of each mask was tested in this study, and while differences are believed to be due to properties of the mask materials, caution must be taken when extrapolating results to other brands. The rapid plastic deformation of the masks limited the number of trials that could be conducted. For some masks it happened so quickly that not even twelve trials could be completed. Furthermore, there is currently no direct link between the head impact metrics analyzed in this study and mTBI. While these are the metrics that are currently used in the field to analyze head injury exposure, there is currently little understanding behind the mechanisms of mTBI which is reflected in the inability to determine a universal metric for mTBI risk. Finally, there is debate as to whether the Hybrid III neck is the proper surrogate for blunt impact injury scenarios. This idea is supported by the work of Heald and Pass, who found that the GSI values when a baseball impacts the side of the head can be an order of magnitude lower for the Hybrid III compared to a cadaveric specimen[21].

More tests will be conducted with a greater number of masks to prevent the limitation of mask damage and provide a greater number of trials for analysis. Also, tests will be conducted with a human cadaver instead of the Hybrid III to provide a more biofidelic model. Finally, there will be an analysis that expands on Schwizer et al. characterizing the foams used in the masks to determine the effect foam characteristics within catcher masks have on head accelerations[19].

The goal of this research was to investigate the impact response of four styles of catcher masks using five common head impact metrics. It was found that overall, the traditional style titanium masks fell in the lowest performance category while the hockey style mask was in the highest performance category. Due to limitations, these results do not provide a direct link to head injury, however they do lay the groundwork for safer mask design.
